# Feasibility of home-based sampling of salivary cortisol and cortisone in healthy adults

**DOI:** 10.1186/s13104-021-05820-4

**Published:** 2021-11-02

**Authors:** Sarah Overgaard Sørensen, Jesper Pedersen, Martin G. Rasmussen, Peter L. Kristensen, Anders Grøntved

**Affiliations:** Research Unit for Exercise Epidemiology, Department of Sports Science and Clinical Biomechanics, Centre of Research in Childhood Health, University of Southern Denmark, Campusvej 55, 5230 Odense, Denmark

**Keywords:** Physiological stress, Saliva, Cortisol, Cortisone, Feasibility

## Abstract

**Objective:**

Salivary cortisol and cortisone are used as biomarkers of physiological stress. Careful sampling of saliva for profiling of awakening response and the diurnal slope can be challenging in free-living environments, and validated sampling protocols are lacking. Therefore, we investigated (1) the level of compliance to a three-day home-based salivary sampling protocol, and (2) the within subject day-to-day variability of cortisol and cortisone outcomes and the required measuring days to obtain high reproducibility.

**Results:**

Nineteen healthy adults (mean age: 42, 50% females) participated. Participants collected in total 434 salivary samples out of 456 scheduled (four samples per day over three consecutive days at two time points). We found high level of compliance to the proposed free-living salivary sampling protocol with 18 (95%) and 16 (84%) participants being compliant to numbers and timing of samples, respectively. The area under the curve for the morning salivary samples and peak-to-bed slope had moderate reproducibility for cortisol and cortisone (intraclass correlation coefficient: 0.51–0.68, and mean coefficient of variation: 14.7%-75.3%). Three-to-four measuring days were required for high reproducibility of the area under the curve for the morning salivary samples and peak-to-bed slope using this free-living salivary sampling protocol.

*Trial registration* Clinical trial registered with www.clinicaltrials.gov (NCT03788525).

**Supplementary Information:**

The online version contains supplementary material available at 10.1186/s13104-021-05820-4.

## Introduction

Salivary cortisol is frequently used as a surrogate for free serum cortisol. Flatter diurnal cortisol slope (DCS) has been associated with poorer emotional health outcomes [1]. The cortisol awakening response (CAR) is positively related to general life stress [2] and alterations of the cortisol awakening response are associated with chronic stress [3]. However, salivary cortisol is rapidly converted to salivary cortisone [4] and cortisone was recently found to be a superior surrogate for free serum cortisol compared to salivary cortisol [5, 6]. Despite this, studies investigating both cortisol and cortisone circadian rhythm patterns in subjects in free-living conditions are lacking [7]. To assess these measures it is necessary to collect salivary samples at several time points and across multiple days [8, 9], which can be challenging when participants are performing the sampling in a daily-life setting [10]. Therefore, it is important to examine participant compliance to a sampling protocol, and to investigate the within subject day-to-day variability of cortisol and cortisone outcomes. This could inform decisions that may help minimize the participant burden and optimize use of resources.

The primary aim of this study was to investigate the level of compliance to a home-based three-day salivary sampling protocol among healthy adults. The secondary aims were to investigate the within subject day-to-day variability of cortisol and cortisone outcomes and estimate the required number of measuring days to obtain acceptable reproducibility, and to inform statistical power calculations for future trials.

## Main text

### Methods

#### Study design and data

The current study uses data from the SCREENS (no abbreviation) pilot trial (www.clinicaltrials.gov (NCT03788525)), which is a parallel-group two-arm cluster randomized trial with no control group carried out in a free-living setting [11]. The SCREENS pilot trial included 12 families (see Additional file 1).

#### Salivary sampling protocol

Participants received instructions and were provided with written information detailing the saliva sampling protocol in a face-to-face meeting with a member of the research team in the participants’ home. Participants were instructed to collect salivary samples on three consecutive days at baseline and follow-up, respectively. Samples were collected immediately upon awakening (S1) i.e. when they woke up and got out of bed, 30 min and 45 min after awakening and once just before bedtime (Additional file 2). Participants were provided with a dual digital timer (S. Brannan Sons Ltd., England) and were instructed to start the timer upon awakening. The timer then rang 30 and 45 min after awakening reminding participants to collect the second and third sample, respectively.

Participants were also instructed not to eat, smoke, exercise, or drink anything but water between the morning samples. Participants were allowed to brush their teeth within the first 20 min after the first salivary sample, and instructed not to drink water after the first 20 min had passed and until they finished the morning sampling routine. In the last 30 min before the evening sample (just before bedtime), participants were instructed not to eat, smoke, exercise, or drink anything other than water.

Samples were collected using Salivette tubes containing a synthetic swab (Starstedt, Nümbrecht, Germany). To collect a sample, participants were instructed to place a swab in their mouths, chew on it lightly for 45–60 s, transfer it back into the tube directly from the mouth and put a pre-labelled sticker on the tube. The samples were stored in a freezer in the home of the participants before they, at the end of the trial, were transported to Slagelse Hospital for storage at −80 °C before laboratory analyses.

#### Analyses of salivary cortisol and cortisone

Salivary cortisol and cortisone were measured using isotope dilution-liquid chromatography-tandem mass spectrometry. Cortisol and cortisone awakening response was both calculated as the difference between the sample collected immediately upon awakening and the sample 30 min later (CAR30) and as the difference between the sample collected immediately upon awakening and the value of the second or third morning samples, whichever was the largest (CARpeak). Cortisol and cortisone awakening response summary indicators were calculated as the area under the curve (CARauc) for the morning samples (upon awakening, 30 min, 45 min after awakening). Diurnal cortisol and cortisone slope was measured as wake-to-bed slopes and peak-to-bed slopes. Wake-to-bed slopes were calculated by subtracting the bedtime sample value from the sample collected immediately upon awakening and divided by the number of hours separating these two samples. Peak-to-bed slopes were calculated by subtracting the bedtime sample from the peak value of the second or third sample in the morning and divided by the number of hours separating these two samples.

#### Checklist

Participants filled in a checklist to report their wake-up time, bedtime, and the time they had taken each sample. The research team manually corrected if a participant had made an obvious typo e.g. in recorded time of wakening or salivary sampling (5 samples). An obvious typo could be if a participant reported salivary collection at 07:00 am, 07:15 am and 07:45 am, but the wake-up time was reported to be 08:00 am. Then, wake-up time was corrected to 07:00 am. The self-reported times were used to measure the level of compliance to the protocol by investigating the timing of the samples according to the wakeup time (Table 1).

**Table 1:** Definition of compliance

Definition	Explanation
Compliant to number of samples	Two complete days out of 3 with 4 samples/day (16 out
	of 24 samples with 8 samples at B and 8 samples at FU)
Compliant to report sample time	Self-reported sample time for the 16 out of 24 samples (8 in B, 8 in FU)
Compliant to timing of S1	Self-reported delay < 10 min from awakening to S1 for 4 days (2 days in B, 2 days in FU)
Compliant to timing of the second	Self-reported collection of morning samples within 1 h
and third morning sample	after awakening for 4 days (2 days in B, 2 days in FU)

The table present the different definitions of compliance to the salivary sampling protocol

*B* baseline, *FU* Follow-up

#### Socioeconomic status

Education levels were coded using the International Standard Classification of Education (ISCED) [12] and used as an indicator of socioeconomic status.

### Statistical analyses

To investigate the reproducibility of cortisol and cortisone concentrations over the three measurement days, within-subject coefficient of variation (CV%) was calculated and intraclass correlation were estimated using linear mixed models with baseline cortisol and cortisone measurements as outcome and subject-id as random effect [13] and corresponds to the expected correlation between cortisol or cortisone outcome measures (i.e. the awakening response) in any pair of days of measurement. Follow-up measurements were used for an additional secondary analysis. The Spearman Brown prophecy formula was used to calculate the necessary sampling days to obtain moderate and high reproducibility [14]. Finally, we conducted a series of power calculations to estimate necessary sample sizes for a future 80% powered parallel group superiority randomized trial for a range of clinically relevant differences (see Additional file 3).

Statistical analyses were performed using the software StataIC (version 16).

### Results

#### Participants

Nineteen adults completed the study. Two participants were noncompliant at one of the six sampling days. The samples from this day were excluded from the analysis of reproducibility (8 samples). The research team checked the objective sleep measurement for these participants before the participant was excluded. The final analytic sample included 354 samples nested in 16 participants (Additional files 4 and 5) and 180 complete days at baseline and 171 days at follow-up. The analyses of compliance were completed before correction and exclusion (n = 19, samples = 434). The raw cortisol data for each included individual at different time points and different days are shown in Additional file 6 to visualize the within and between subject variability.

#### Compliance

As shown in Table 2, 18 (95%) participants were compliant to number of samples according to the definition. A total of 16 (84%) participants were compliant to reporting the salivary sampling time in the checklist for 16 or more samples. Similarly, 16 (84%) participants were compliant to the timing of all morning samples. Higher demands to complete data (3 days) resulted in less compliant participants. A small difference was found in the number of compliant participants at baseline and follow-up when analyzing all three days.

**Table 2:** Compliance to the salivary sampling protocol

	Total	Baseline	Follow-up
Number of samples, n(%)			
1 day in B and FU	18 (94.74)	18 (94.74)	18 (94.74)
2 days in B and FU*	18 (94.74)	18 (94.74)	18 (94.74)
3 days in B and FU	15 (78.95)	15 (78.95)	17 (89.47)
Report sample time, n(%)			
1 day in B and FU	16 (84.21)	16 (84.21)	17 (89.47)
2 days in B and FU*	16 (84.21)	16 (84.21)	16 (84.21)
3 days B and FU	10 (52.63)	13 (68.42)	12 (63.16)
Compliant to timing of S1, n(%)			
1 day in B and FU	16 (84.21)	16 (84.21)	17 (89.47)
2 days in B and FU*	16 (84.21)	16 (84.21)	16 (84.21)
3 days in B and FU	10 (52.63)	12 (63.16)	12 (63.16)
Compliant to timing of the second and third sample, n(%)			
1 day in B and FU	16 (84.21)	16 (84.21)	16 (84.21)
2 days in B and FU*	16 (84.21)	16 (84.21)	16 (84.21)
3 days in B and FU	11 (57.89)	14 (73.68)	12 (63.16)

Total represents the number of participants that are compliant at both baseline and follow-up before exclusion

Data are presented as numbers (percentage)

*B* baseline, *FU* follow-up, *S1* the salivary sample collected immediately upon awakening

*: Corresponds to the definition of compliance (Table 1)

#### Reproducibility of cortisol and cortisone

The within-subject CV% for samples obtained on comparable time-points on different days were moderate-to-large (mean CV% = 14.7%-75.3%, Table 3 and Additional file 7). Analyses indicated moderate within-subject reproducibility for the area under the curve for the morning samples and peak-to-bed slope for both cortisol and cortisone and CARpeak for cortisone at baseline (Table 3). The rest of the intraclass correlation coefficients indicated high within-subject day-to-day variability. The within and between subject reproducibility for the area under the curve for the morning salivary samples and peak-to-bed slope for cortisol at baseline and follow-up are also visualized graphically in Additional file 8. The predicted number of measurement days needed to obtain high reproducibility (intraclass correlation coefficients = 0.80) was three days for peak-to-bed slope for both cortisol and cortisone and 4 days for the area under the curve for the morning salivary samples for cortisol and cortisone. To obtain moderate reproducibility (intraclass correlation coefficients = 0.70) for the area under the curve for the morning salivary samples for cortisol and cortisone and peak-to-bed cortisone slope the samples need to be collected over two days. One day of samples for peak-to-bed cortisol slope is needed to obtain an intraclass correlation coefficients value of 0.70. One day of samples is enough to obtain intraclass correlation coefficients values of 0.60 for the area under the curve for the morning salivary samples and peak-to-bed slope for cortisol and cortisone. The analyses indicated fairly similar results when they were based on follow-up measurements (Additional file 9).

**Table 3:** Intraclass correlation coefficients (ICC) and required measuring days based on baseline measurements

	Mean CV%	ICC	Days to obtain	Days to obtain	Days to obtain
			ICC = 0.60	ICC = 0.70	ICC = 0.80
Cortisol					
S1	31.9	0.41	2	3	6
CAR					
CAR30 (30 min—S1)	75.3	0.17	3	4	7
CARpeak (Peak cortisol—S1)	52.3	0.17	2	3	5
CARauc	20.1	0.56	1	2	4
DCS					
Wake-to-bed slope	59.7	0.35	3	4	7
Peak-to-bed slope	31.9	0.51	1	1	3
Cortisone					
S1	17.1	0.28	4	6	10
CAR					
CAR30 (30 min—S1)	49.0	0.29	4	6	10
CARpeak (Peak cortisol—S1)	44.8	0.60	1	2	3
CARauc	14.7	0.57	1	2	4
DCS					
Wake-to-bed slope	37.5	0.18	7	11	18
Peak-to-bed slope	24.1	0.68	1	2	3

The table present the coefficient of variation (%) and intraclass correlation coefficient (ICC) for concentrations of cortisol and cortisone within the same participant at baseline and the required number of measuring days to obtain ICC values of 0.60, 0.70 and 0.80

*S1* The salivary sample collected immediately upon awakening, *CAR* Cortisol (or cortisone) awakening response, *CAR30* Difference between S1 and the salivary sample 30 min later, *CARpeak* Difference between S1 and the value of the second or third morning salivary sample, whichever was the largest; *CARauc* The area under the curve for the morning salivary samples, *DCS* Diurnal cortisol (or cortisone) slope

#### Necessary sample size in a future study

According to our power calculations, minimally required sample sizes for future parallel group superiority randomized trials are 56 to 162, 36 to 58 and 26 to 42 participants to be able to detect a small (Cohen’s d = 0.3), moderate (Cohen’s d = 0.5) and large (Cohen’s d = 0.6) effect size, respectively (depending on the cortisol and cortisone outcome) (Additional file 3).

### Discussion

The main finding of the current study was the high level of compliance to the home-based three-day salivary sampling protocol that was designed to balance the burden on participants and acquisition of valid data. Although a 5–15% amount of missing data due to lack of compliance to the assessment protocol is unlikely to be a serious threat to the internal validity of a future study, additional measures that may improve participant compliance may be needed. The protocol could be improved by validating the timing of the salivary sampling using objective sleep measurements (i.e. a wrist-worn device) to record the wake-up time. Furthermore, reporting errors may be eliminated by using an app to record timing.

The area under the curve for the morning salivary samples and peak-to-bed slope showed moderate day-to-day reproducibility over three days, which are in line with findings in the literature [15–17]. The current study found high day-to-day variability and low reproducibility for the few remaining measurements of cortisol and cortisone, which also consist with the literature [15, 17, 18]. Besides, a previous study by Bakusic et al. confirms a high-day-to-day variability of both cortisol and cortisone. However, this study found higher reproducibility for cortisone compared to cortisol which may indicate that cortisone may be a more stable measure compared to cortisol [7]. In the current study we found no evidence of a difference in reproducibility between cortisol and cortisone.

The results showed that samples should be collected over at least 3–4 and 1–2 days if high or moderate reproducibility respectively is minimally desirable.

The study possesses several strength including the carful collection of participants reported timing of samples, bed-and wake times, and the possibility of cross-checking self-reported wake-time with objective sleep measurement made it possible to obtain a reasonable investigation of the feasibility of our sampling protocol. Furthermore, a strength of this study was the use of isotope dilution-liquid chromatography-tandem mass spectrometry which has high accuracy and specificity.

### Conclusion

In conclusion, we found high levels of compliance to the home-based salivary sampling protocol in a sample of healthy adults. The within subject day-to-day variability was fairly high for all cortisol and cortisone outcomes investigated. A two-day sampling protocol appears to yield the right balance between resource use and minimization of participant burden if a moderate reproducibility is deemed sufficient in a study.

## Limitations

The assessments of compliance to the timing of the samples were made based on self-report. Also, the relatively small number of participants may limit the external validity of the study. Finally, it was only possible to use the objective sleep and wake time to validate if the self-reported waking time was accurately reported in some cases.

## Supplementary Information

**Additional file 1.** Flow chart of the recruitment. The figure shows a graphic presentation of the recruitment process.

**Additional file 2.** Protocol for sampling collection. The figure presents the protocol for sampling collection across 3 days.

**Additional file 3: Table S1.** Change from baseline to follow-up for all participants in the SCREENS pilot trial. Data are presented as means with SD. **Figure S1.** Estimated sample size for CARauc cortisol. Sample size estimation for a future parallel group randomized controlled superiority trial (randomized in 1:1 ratio) powered at 80% with a range of estimated relevant differences between groups. Estimations were based on the standard deviation (SD) of- and the correlation between the outcome at baseline and follow-up respectively with alpha = 0.05. Calculations were carried out based on using the follow-up measure as outcome with adjustment for the outcome at baseline (similar to an analysis of co-variance). **Figure S2**. Estimated sample size for CARauc cortisone. Sample size estimation for a future parallel group randomized controlled superiority trial (randomized in 1:1 ratio) powered at 80% with a range of estimated relevant differences between groups. Estimations were based on the standard deviation (SD) of- and the correlation between the outcome at baseline and follow-up respectively with alpha = 0.05. Calculations were carried out based on using the follow-up measure as outcome with adjustment for the outcome at baseline (similar to an analysis of co-variance). **Figure S3.** Estimated sample size for peak-to-bed cortisol slope. Sample size estimation for a future parallel group randomized controlled superiority trial (randomized in 1:1 ratio) powered at 80% with a range of estimated relevant differences between groups. Estimations were based on the standard deviation (SD) of- and the correlation between the outcome at baseline and follow-up respectively with alpha = 0.05. Calculations were carried out based on using the follow-up measure as outcome with adjustment for the outcome at baseline (similar to an analysis of co-variance). **Figure S4.** Estimated sample size for peak-to-bed cortisone slope. Sample size estimation for a future parallel group randomized controlled superiority trial (randomized in 1:1 ratio) powered at 80% with a range of estimated relevant differences between groups. Estimations were based on the standard deviation (SD) of- and the correlation between the outcome at baseline and follow-up respectively with alpha = 0.05. Calculations were carried out based on using the follow-up measure as outcome with adjustment for the outcome at baseline (similar to an analysis of co-variance).

**Additional file 4.** Flow chart of exclusion from the analyses. The figure shows a graphic presentation of the exclusion process before the analyses. B = Baseline; FU = Follow-up; CAR = Cortisol awakening response; DCS = Diurnal cortisol slope; ICC = Intraclass correlation.

**Additional file 5.** Characteristics of participants. The table present characteristics of participants at baseline. Data are presented as means with SD when data were approximately normal distributed. Data were calculated as medians with 25th and 75th percentiles for non-parametric distribution of data. Categorized data are presented as proportions. Measuring day are presented as the proportion of salivary samples taken on weekdays.

**Additional file 6: Figure S1.** Raw data for the morning cortisol samples for each individual represented by a colored marker and line. More lines with the same color represent multiple days within individuals. **Figure S2.** Raw data for the first morning cortisol sample and the evening sample (the diurnal slope) for each individual represented by a colored marker and line. More lines with the same color represent multiple days within individuals.

**Additional file 7: Figure S1.** Box plots showing the within-subject CV% for cortisol samples obtained on comparable time-points on different days at baseline. **Figure S2.** Box plots showing the within-subject CV% for cortisone samples obtained on comparable time-points on different days at baseline.

**Additional file 8: Figure S1.** Within and between subject reproducibility for CARauc for cortisol at baseline. **Figure S2.** Within and between subject reproducibility for CARauc for cortisol at follow-up. **Figure S3.** Within and between subject reproducibility for peak-to-bed cortisol slope at baseline. **Figure S4.** Within and between subject reproducibility for peak-to-bed cortisol slope at follow-up.

**Additional file 9.** Intraclass correlation coefficients (ICC) for different cortisol and cortisone measurements and required measuring day based on follow-up values. The table present the intraclass correlation coefficient (ICC) for concentrations of cortisol and cortisone within the same participant and the required number of measuring days to obtain ICC values of 0.60, 0.70 and 0.80.

## Data Availability

The datasets used and/or analyzed during the current study are available from the corresponding author on reasonable request.

## Abbreviations

B: Baseline
CAR: Cortisol (or cortisone) awakening response
CAR30: Difference between S1 and the salivary sample 30 min later
CARauc: The area under the curve for the morning salivary samples (S1, 30 min, 45 min after awakening)
CARpeak: Difference between S1 and the value of the second or third morning salivary sample, whichever was the largest
DCS: Diurnal cortisol (or cortisone) slope
FU: Follow-up
ICC: Intraclass correlation coefficient
S1: Salivary sample collected immediately upon awakening
