# Supplementary figures and images for: Feasibility of home-based sampling of salivary cortisol and cortisone in healthy adults
Source: BMC Res Notes. 2021 Nov 2;14:406. doi: 10.1186/s13104-021-05820-4 (PMC8561883; doi:10.1186/s13104-021-05820-4)

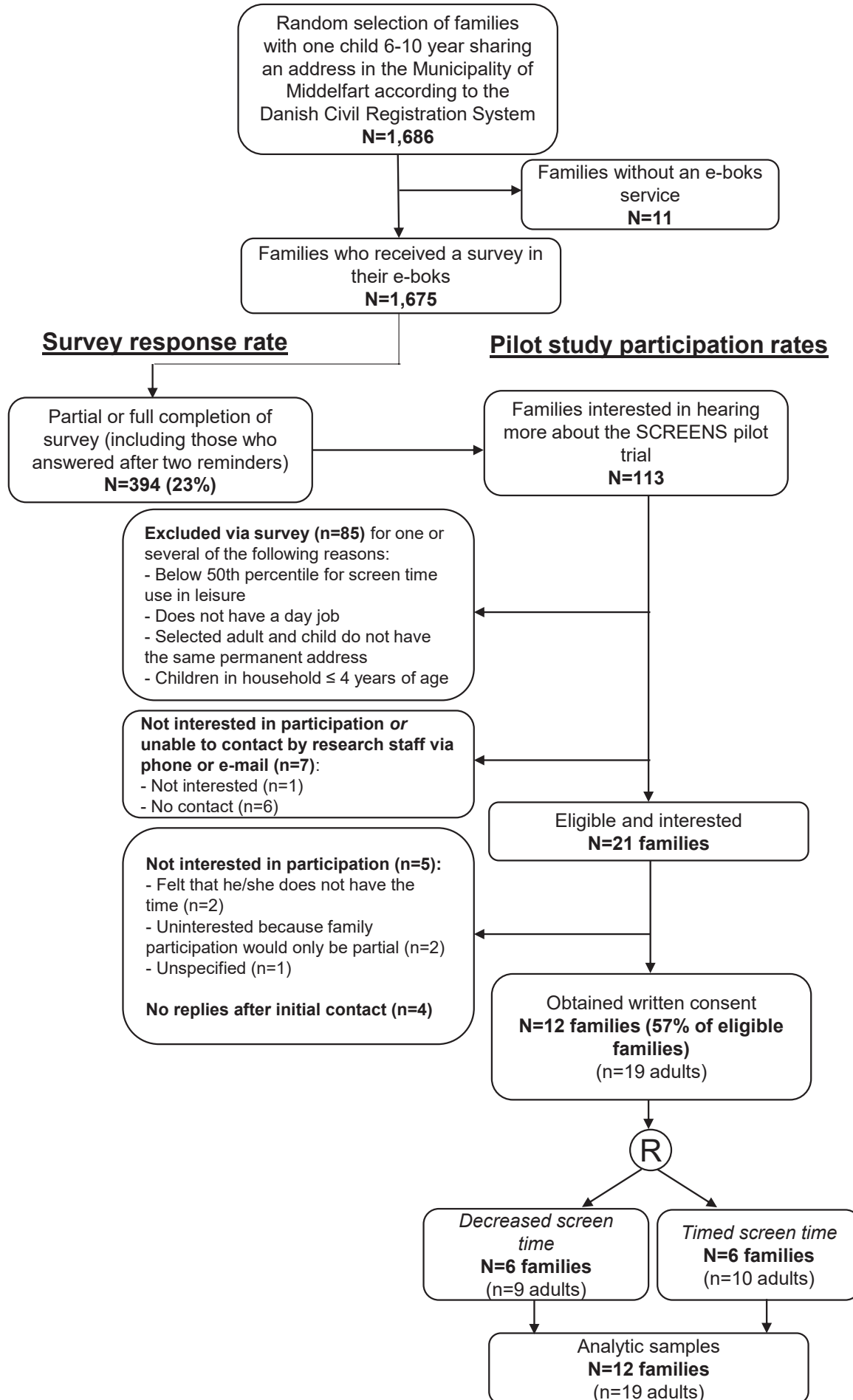

Supplement: Supplementary file 1 — Additional file 1. Flow chart of the recruitment. The figure shows a graphic presentation of the recruitment process. [file 13104_2021_5820_MOESM1_ESM.pdf]

Additional file 2: Protocol for sampling collection


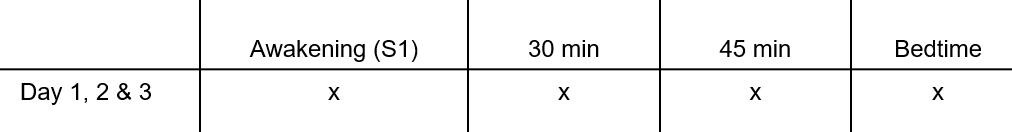

Supplement: Supplementary file 2 — Additional file 2. Protocol for sampling collection. The figure presents the protocol for sampling collection across 3 days. [file 13104_2021_5820_MOESM2_ESM.docx]
