# Supplementary material for: Feasibility of home-based sampling of salivary cortisol and cortisone in healthy adults
Source: BMC Res Notes. 2021 Nov 2;14:406. doi: 10.1186/s13104-021-05820-4 (PMC8561883; doi:10.1186/s13104-021-05820-4)
Supplement: Supplementary file 3 — Additional file 3: Table S1. Change from baseline to follow-up for all participants in the SCREENS pilot trial. Data are presented as means with SD. Figure S1. Estimated sample size for CARauc cortisol. Sample size estimation for a future parallel group randomized controlled superiority trial (randomized in 1:1 ratio) powered at 80% with a range of estimated relevant differences between groups. Estimations were based on the standard deviation (SD) of- and the correlation between the outcome at baseline and follow-up respectively with alpha = 0.05. Calculations were carried out based on using the follow-up measure as outcome with adjustment for the outcome at baseline (similar to an analysis of co-variance). Figure S2. Estimated sample size for CARauc cortisone. Sample size estimation for a future parallel group randomized controlled superiority trial (randomized in 1:1 ratio) powered at 80% with a range of estimated relevant differences between groups. Estimations were based on the standard deviation (SD) of- and the correlation between the outcome at baseline and follow-up respectively with alpha = 0.05. Calculations were carried out based on using the follow-up measure as outcome with adjustment for the outcome at baseline (similar to an analysis of co-variance). Figure S3. Estimated sample size for peak-to-bed cortisol slope. Sample size estimation for a future parallel group randomized controlled superiority trial (randomized in 1:1 ratio) powered at 80% with a range of estimated relevant differences between groups. Estimations were based on the standard deviation (SD) of- and the correlation between the outcome at baseline and follow-up respectively with alpha = 0.05. Calculations were carried out based on using the follow-up measure as outcome with adjustment for the outcome at baseline (similar to an analysis of co-variance). Figure S4. Estimated sample size for peak-to-bed cortisone slope. Sample size estimation for a future parallel group randomized control [file 13104_2021_5820_MOESM3_ESM.pdf]

Additional file 3: Sample size estimation for a future parallel group randomized controlled superiority trial (randomized in 1:1 ratio) powered at 80 % with a range of estimated relevant differences between groups. Estimations were based on the standard deviation (SD) of- and the correlation between the outcome at baseline and follow-up respectively with alpha=0.05 (see Table below). Calculations were carried out based on using the follow-up measure as outcome with adjustment for the outcome at baseline (similar to an analysis of co-variance).

Table: Change from baseline to follow-up for all participants in the SCREENS pilot trial

	Baseline (SD)	Follow-up (SD)	Change (SD)	Correlation
CARauc cortisol (nmol/L x minutes)	6.84 (2.97)	7.14 (3.02)	0.85 (1.46)	0.89
CARauc cortisone (nmol/L x minutes)	26.85 (7.47)	27.53 (6.81)	0.97 (3.69	0.81
Peak-to-bed slope cortisol (nmol/L)	0.65 (0.32)	0.64 (0.33)	0.02 (0.21)	0.73
Peak-to-bed slope cortisone (nmol/L)	2.28 (0.73)	2.30 (0.82)	0.05 (0.39)	0.81

Data are presented as means with SD.

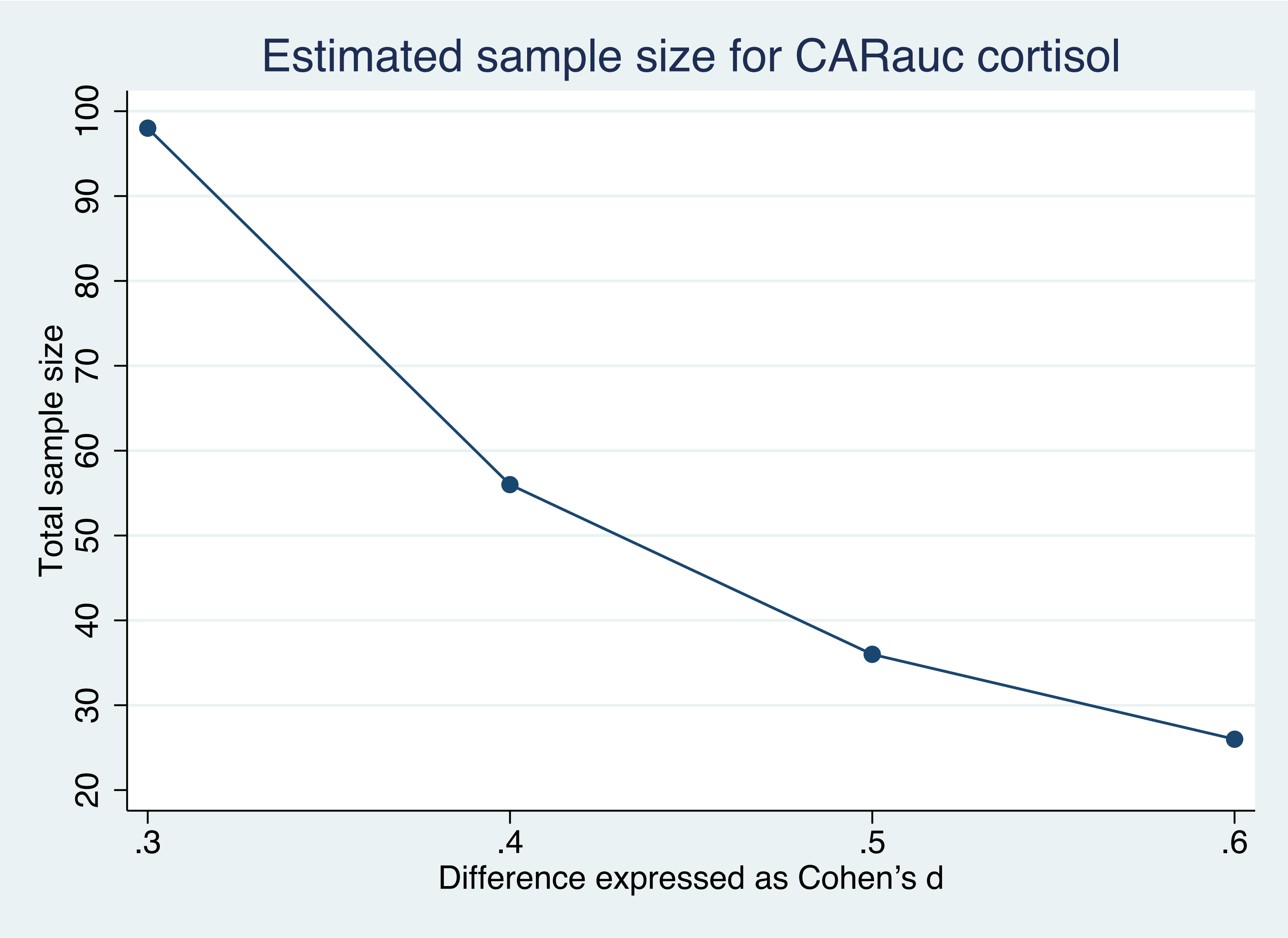

Estimated sample size for CARauc cortisol
Total sample size
100
90
80
70
60
50
40
30
20
.3
.4
.5
.6
Difference expressed as Cohen's d

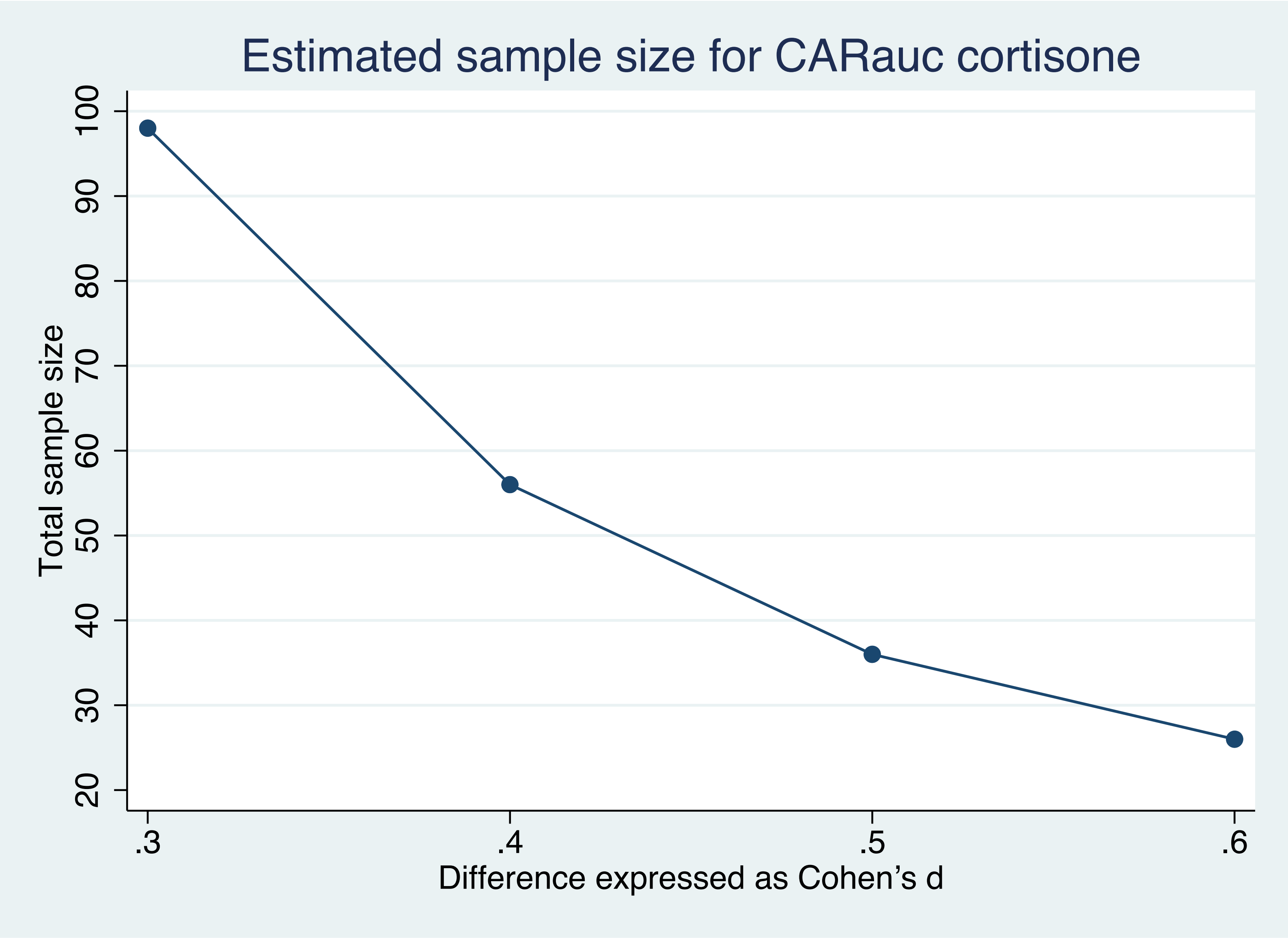

Estimated sample size for CARauc cortisone
Total sample size
20
30
40
50
60
70
80
90
100
.3
.4
.5
.6
Difference expressed as Cohen's d

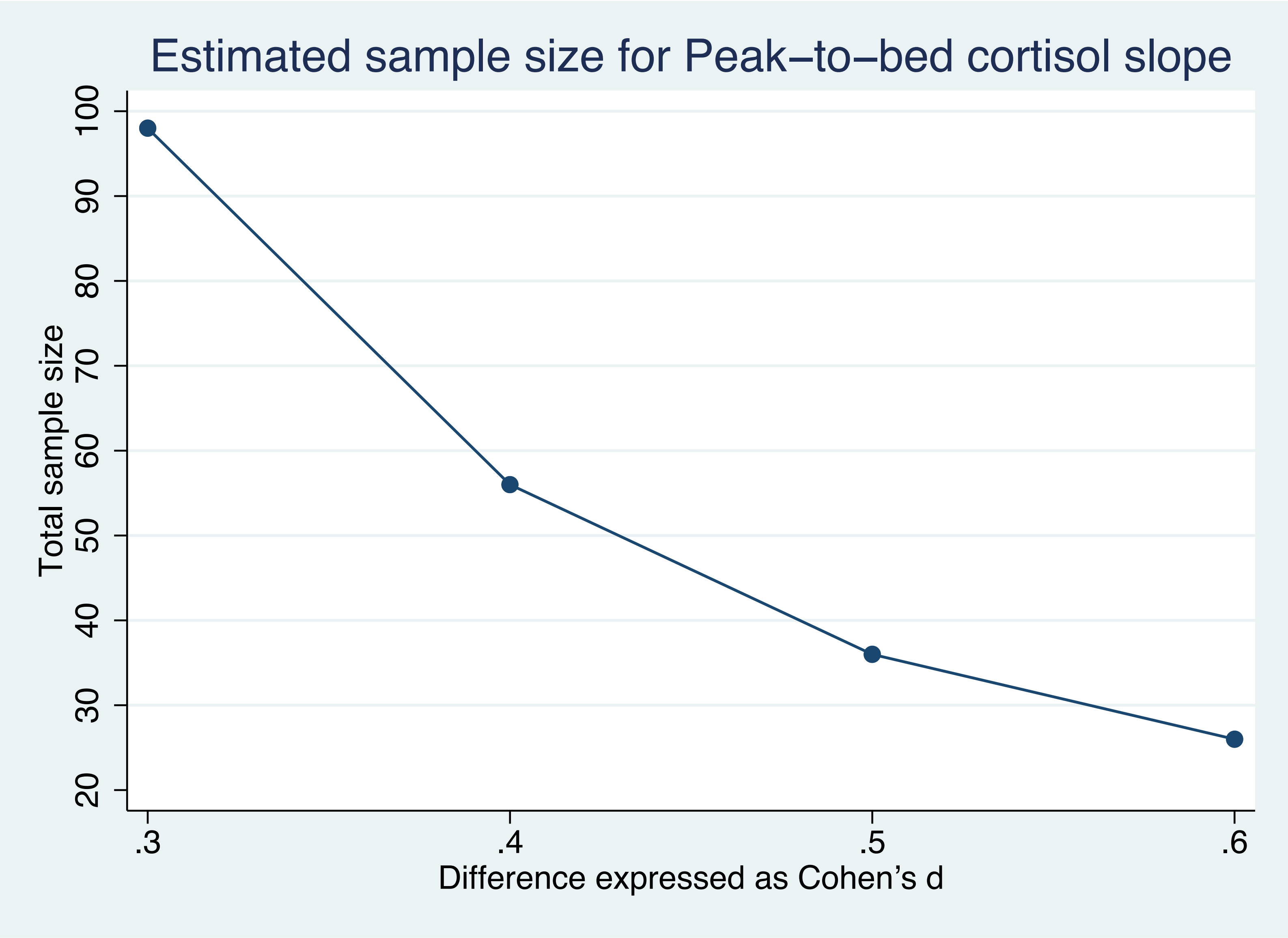
Estimated sample size for Peak–to–bed cortisol slope
Total sample size
100
90
80
70
60
50
40
30
20
.3
.4
.5
.6
Difference expressed as Cohen's d

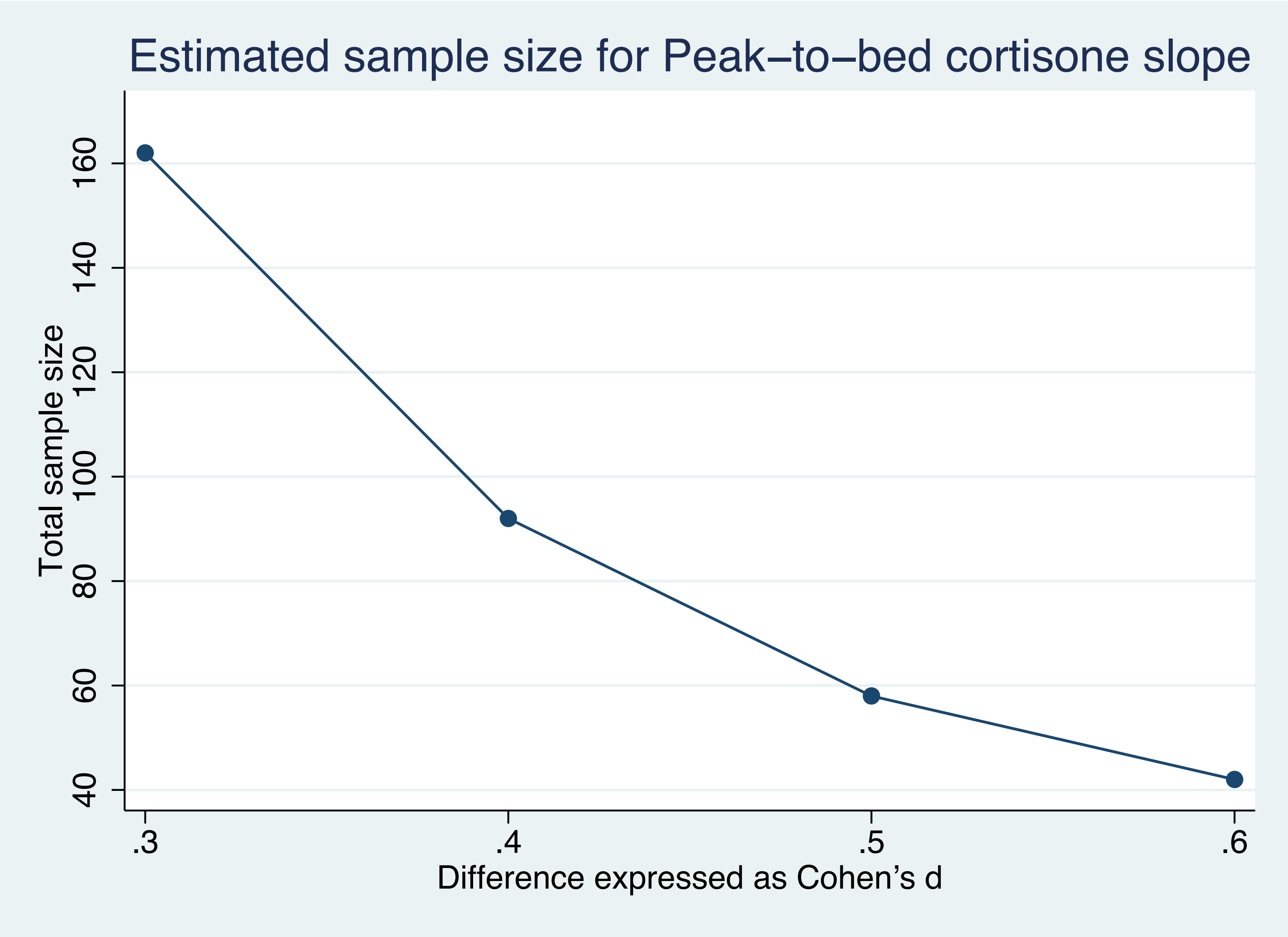
Estimated sample size for Peak–to–bed cortisone slope
Total sample size
40
60
80
100
120
140
160
.3
.4
.5
.6
Difference expressed as Cohen's d
