# Supplementary material for: Feasibility of home-based sampling of salivary cortisol and cortisone in healthy adults
Source: BMC Res Notes. 2021 Nov 2;14:406. doi: 10.1186/s13104-021-05820-4 (PMC8561883; doi:10.1186/s13104-021-05820-4)
Supplement: Supplementary file 4 — Additional file 4. Flow chart of exclusion from the analyses. The figure shows a graphic presentation of the exclusion process before the analyses. B = Baseline; FU = Follow-up; CAR = Cortisol awakening response; DCS = Diurnal cortisol slope; ICC = Intraclass correlation. [file 13104_2021_5820_MOESM4_ESM.pdf]

Additional file 4: Flow chart of exclusion from the analyses

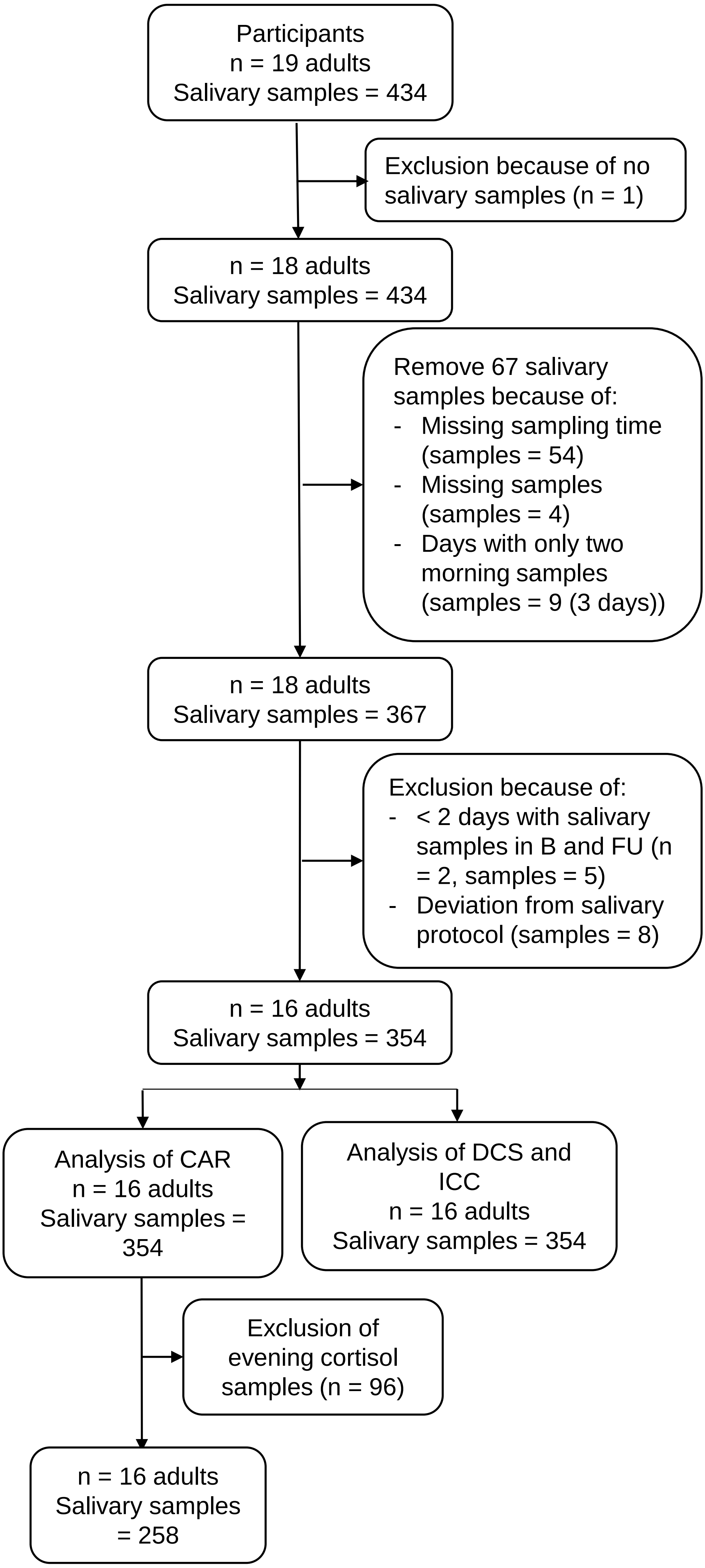

The figure shows a graphic presentation of the exclusion process before the analyses.
B = Baseline; FU = Follow-up; CAR = Cortisol awakening response; DCS = Diurnal cortisol slope; ICC = Intraclass correlation.
