# Supplementary material for: Feasibility of home-based sampling of salivary cortisol and cortisone in healthy adults
Source: BMC Res Notes. 2021 Nov 2;14:406. doi: 10.1186/s13104-021-05820-4 (PMC8561883; doi:10.1186/s13104-021-05820-4)
Supplement: Supplementary file 5 — Additional file 5. Characteristics of participants. The table present characteristics of participants at baseline. Data are presented as means with SD when data were approximately normal distributed. Data were calculated as medians with 25th and 75th percentiles for non-parametric distribution of data. Categorized data are presented as proportions. Measuring day are presented as the proportion of salivary samples taken on weekdays. [file 13104_2021_5820_MOESM5_ESM.docx]

Additional file 5: Characteristics of participants

|  | | |
| --- | --- | --- |
| n | | 16 |
| Age (years) | | 42 (40-46) |
| Sex (number of females (%)) | | 8 (50.0) |
| ISCED (%) | |  |
|  | 1-2 | 6.78 |
|  | 3-4 | 29.38 |
|  | 5-6 | 43.50 |
|  | 7-8 | 20.34 |
| Measuring days (%weekdays) | | 66.11 |
| Cortisol | | 8.27 (5.74-11.54) |
| S1 (nmol/L) | | 5.75 (2.58) |
| 30 min following awakening (nmol/L) | | 10.74 (7.61-14.21) |
| 45 min following awakening (nmol/L) | | 9.70 (6.32-13-42) |
| Bedtime (nmol/L) | | 1.24 (0.81-1.68) |
| CAR (nmol/L) | |  |
|  | CAR30 (30 min - S1) | 5.35 (1.53-8.49) |
|  | CARpeak (Peak cortisol - S1) | 6.95 (3.76-10.72) |
| CARauc | | 6.93 (3.03) |
| Diurnal cortisol slope | |  |
|  | Wake-to-bed slope | 0.26 (0.17) |
|  | Peak-to-bed slope | 0.70 (0.38) |
| Cortisone | | 35.83 (13.11) |
| S1 (nmol/L) | | 26.33 (7.04) |
| 30 min following awakening (nmol/L) | | 41.62 (33.36-49.11) |
| 45 min following awakening (nmol/L) | | 39.34 (34.30-46.83) |
| Bedtime (nmol/L) | | 7.18 (5.22-9.16) |
| CAR (nmol/L) | |  |
|  | CAR30 (30 min - S1) | 14.22 (8.58) |
|  | CARpeak (Peak cortisol - S1) | 17.58 (10.23-23.27) |
| CARauc | | 27.39 (22.81-30.73) |
| Diurnal cortisol slope | |  |
|  | Wake-to-bed slope | 1.12 (0.45) |
|  | Peak-to-bed slope | 2.25 (0.84) |
