# Supplementary material for: Feasibility of home-based sampling of salivary cortisol and cortisone in healthy adults
Source: BMC Res Notes. 2021 Nov 2;14:406. doi: 10.1186/s13104-021-05820-4 (PMC8561883; doi:10.1186/s13104-021-05820-4)
Supplement: Supplementary file 6 — Additional file 6: Figure S1. Raw data for the morning cortisol samples for each individual represented by a colored marker and line. More lines with the same color represent multiple days within individuals. Figure S2. Raw data for the first morning cortisol sample and the evening sample (the diurnal slope) for each individual represented by a colored marker and line. More lines with the same color represent multiple days within individuals. [file 13104_2021_5820_MOESM6_ESM.pdf]

Additional file 6: Raw cortisol data for each individual at different time points and different days to visualize the within and between subject variability

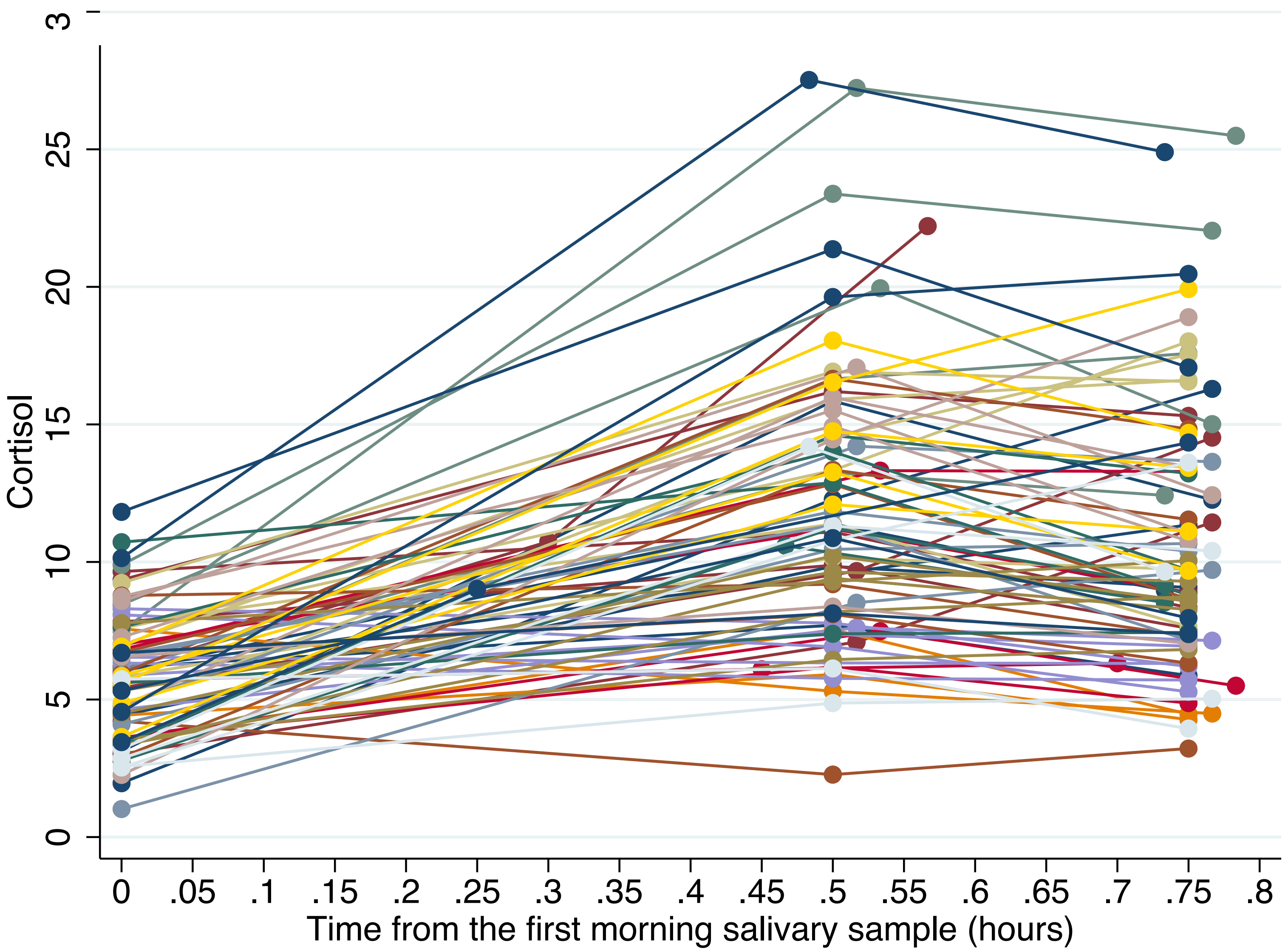
Cortisol
0
5
10
15
20
25
3
0
.05
.1
.15
.2
.25
.3
.35
.4
.45
.5
.55
.6
.65
.7
.75
.8
Time from the first morning salivary sample (hours)

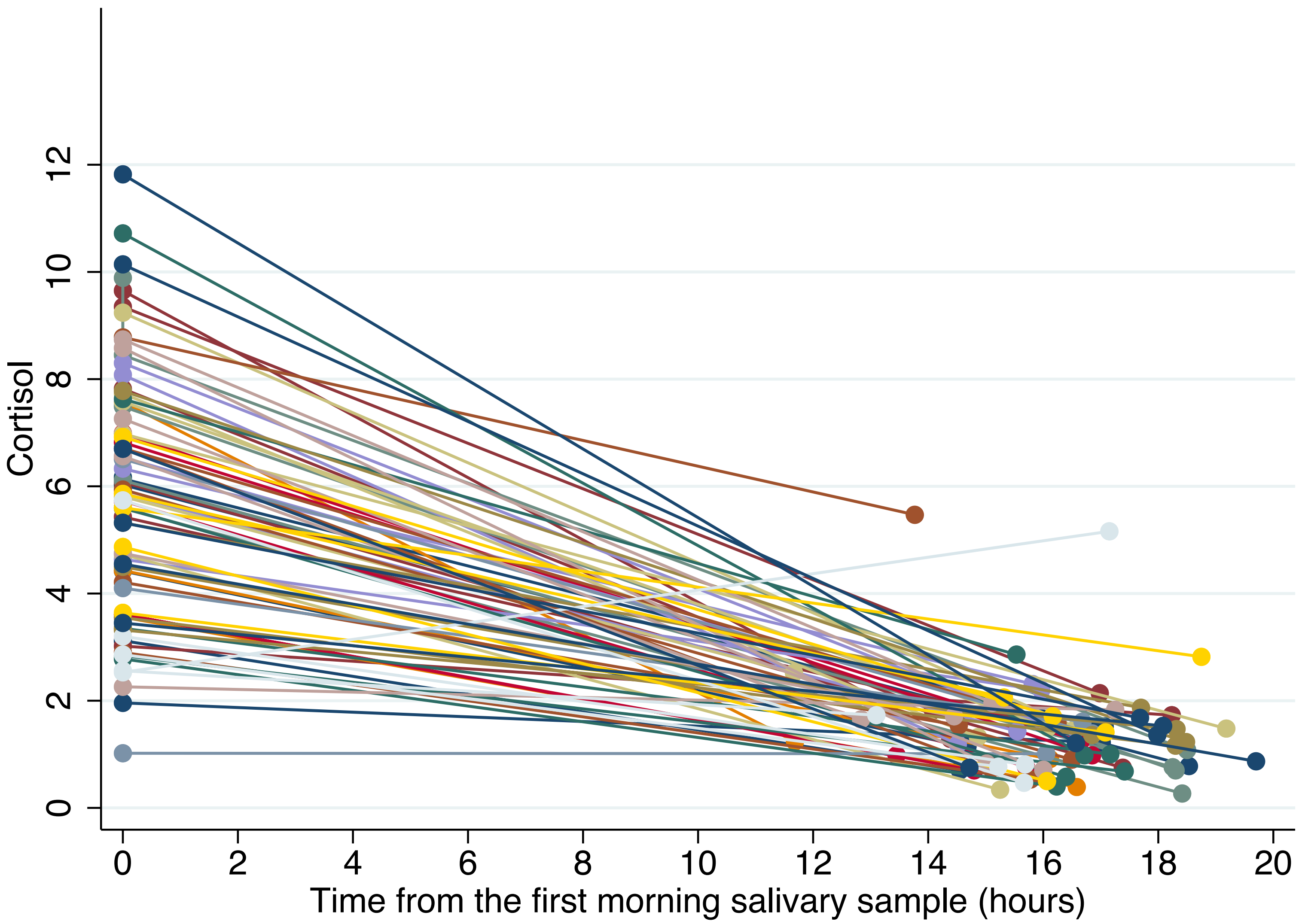

Cortisol
0
2
4
6
8
10
12
0
2
4
6
8
10
12
14
16
18
20
Time from the first morning salivary sample (hours)
