# Supplementary material for: Feasibility of home-based sampling of salivary cortisol and cortisone in healthy adults
Source: BMC Res Notes. 2021 Nov 2;14:406. doi: 10.1186/s13104-021-05820-4 (PMC8561883; doi:10.1186/s13104-021-05820-4)
Supplement: Supplementary file 7 — Additional file 7: Figure S1. Box plots showing the within-subject CV% for cortisol samples obtained on comparable time-points on different days at baseline. Figure S2. Box plots showing the within-subject CV% for cortisone samples obtained on comparable time-points on different days at baseline. [file 13104_2021_5820_MOESM7_ESM.pdf]

Additional file 7: The within-subject coefficient of variation (CV%) for samples obtained on comparable time-points on different days at baseline

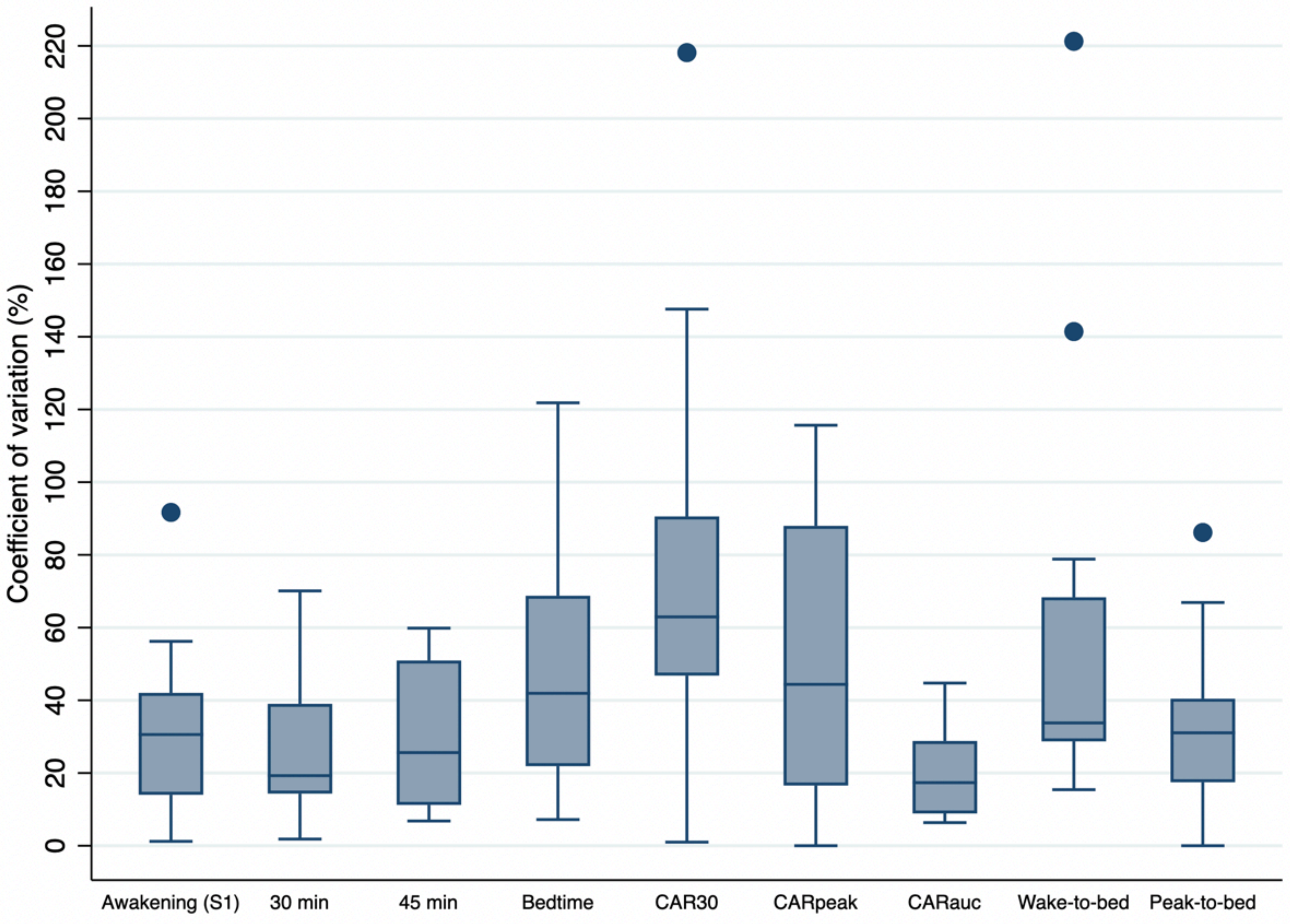
Coefficient of variation (%)
0
20
40
60
80
100
120
140
160
180
200
220
Awakening (S1)
30 min
45 min
Bedtime
CAR30
CARpeak
CARauc
Wake-to-bed
Peak-to-bed

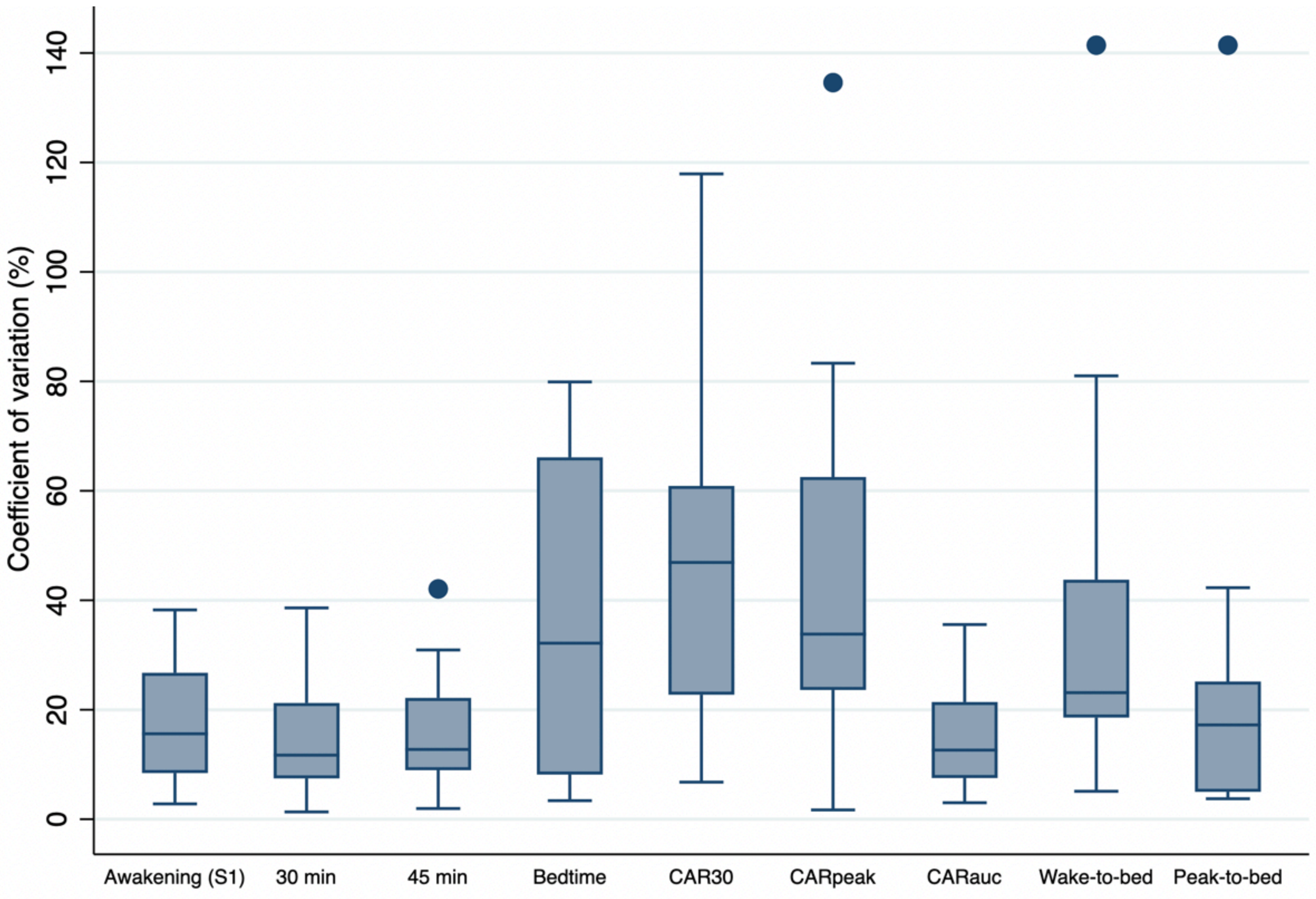
Coefficient of variation (%)
0
20
40
60
80
100
120
140
Awakening (S1)
30 min
45 min
Bedtime
CAR30
CARpeak
CARauc
Wake-to-bed
Peak-to-bed
