# Supplementary material for: Feasibility of home-based sampling of salivary cortisol and cortisone in healthy adults
Source: BMC Res Notes. 2021 Nov 2;14:406. doi: 10.1186/s13104-021-05820-4 (PMC8561883; doi:10.1186/s13104-021-05820-4)
Supplement: Supplementary file 8 — Additional file 8: Figure S1. Within and between subject reproducibility for CARauc for cortisol at baseline. Figure S2. Within and between subject reproducibility for CARauc for cortisol at follow-up. Figure S3. Within and between subject reproducibility for peak-to-bed cortisol slope at baseline. Figure S4. Within and between subject reproducibility for peak-to-bed cortisol slope at follow-up. [file 13104_2021_5820_MOESM8_ESM.pdf]

Additional file 8: The within and between subject reproducibility for CARauc and peak-to-bed slope for cortisol at baseline and follow-up

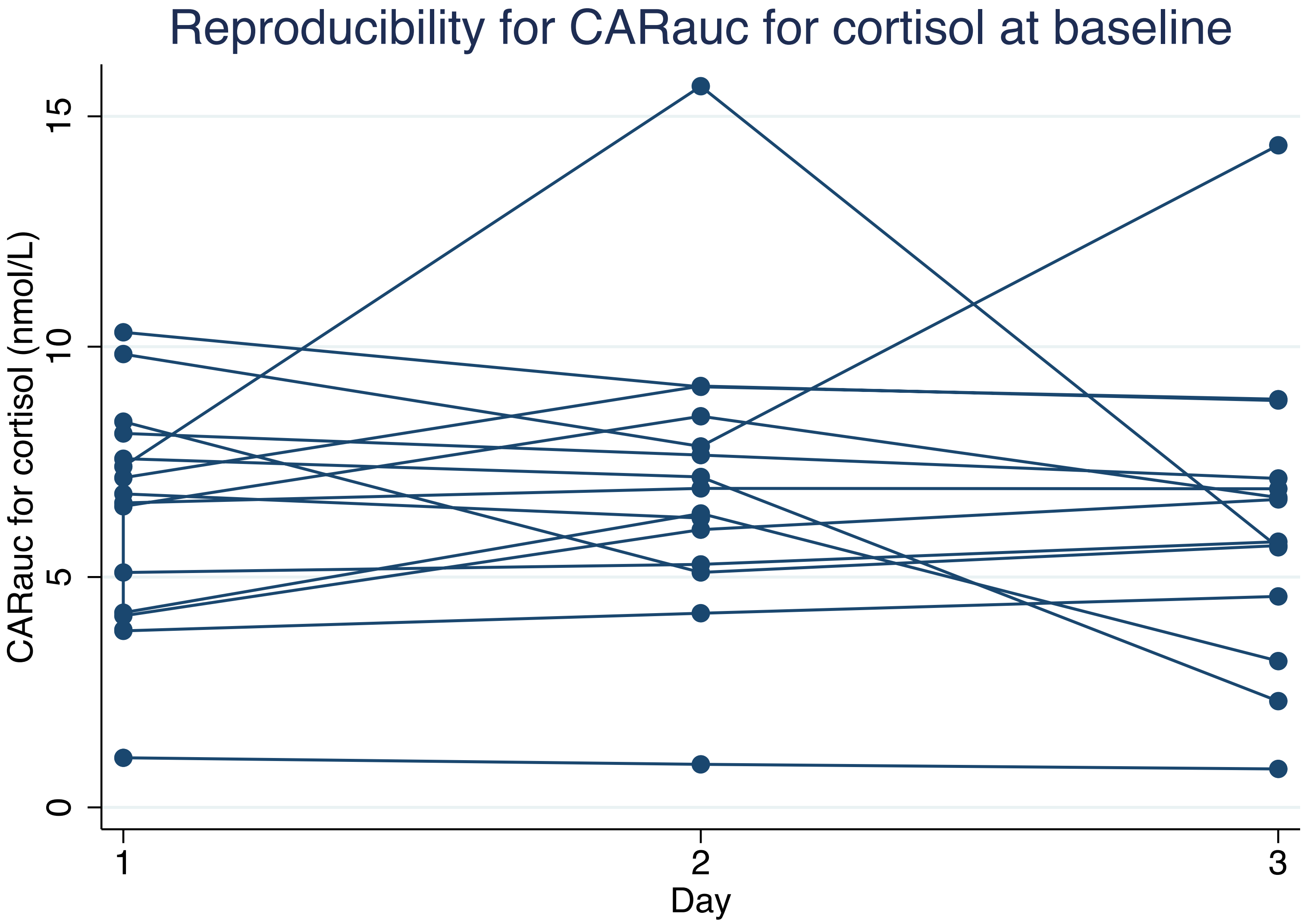
Reproducibility for CARauc for cortisol at baseline
CARauc for cortisol (nmol/L)
0
5
10
15
1
2
3
Day

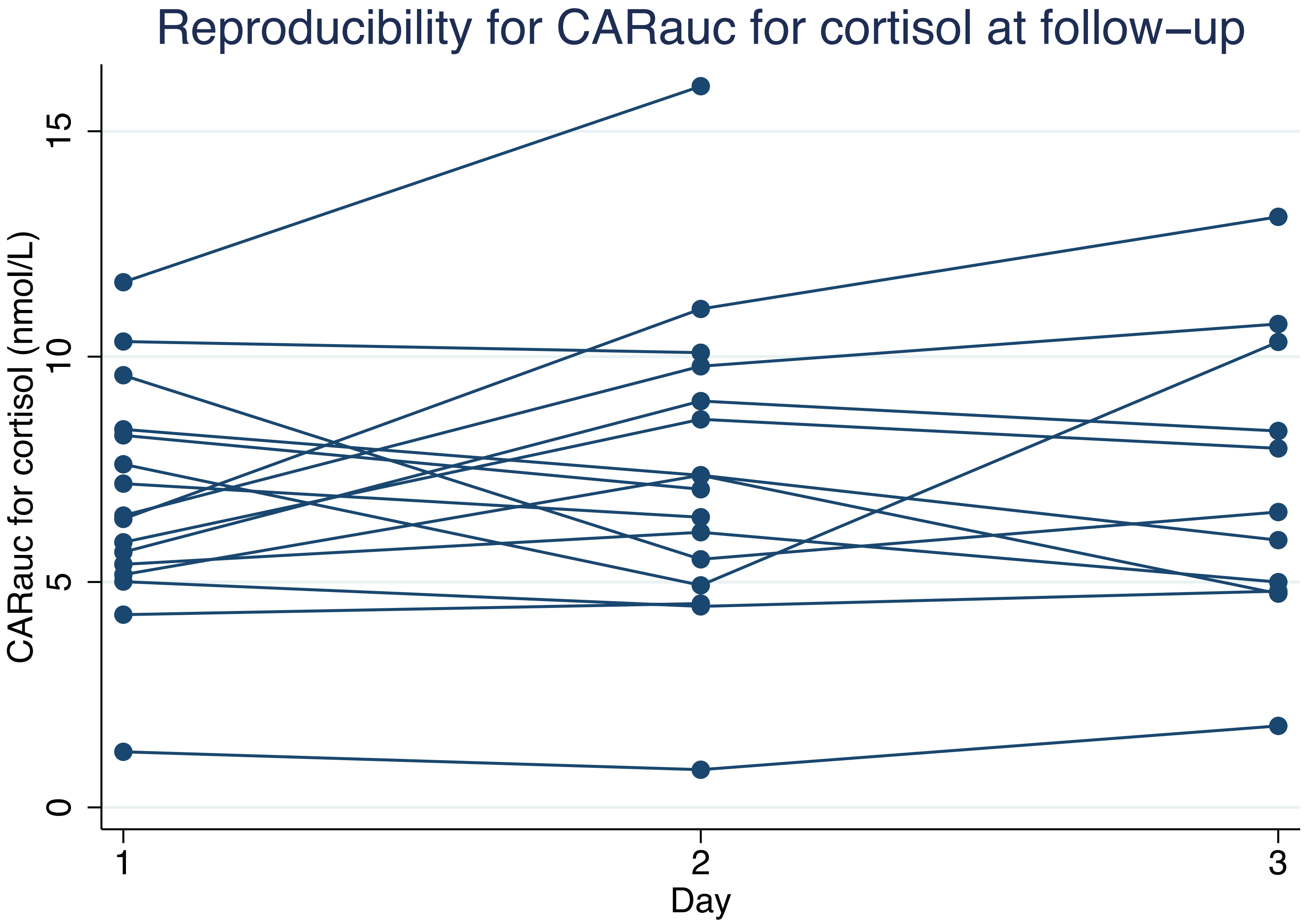

Reproducibility for CARauc for cortisol at follow−up
CARauc for cortisol (nmol/L)
0
5
10
15
1
2
3
Day

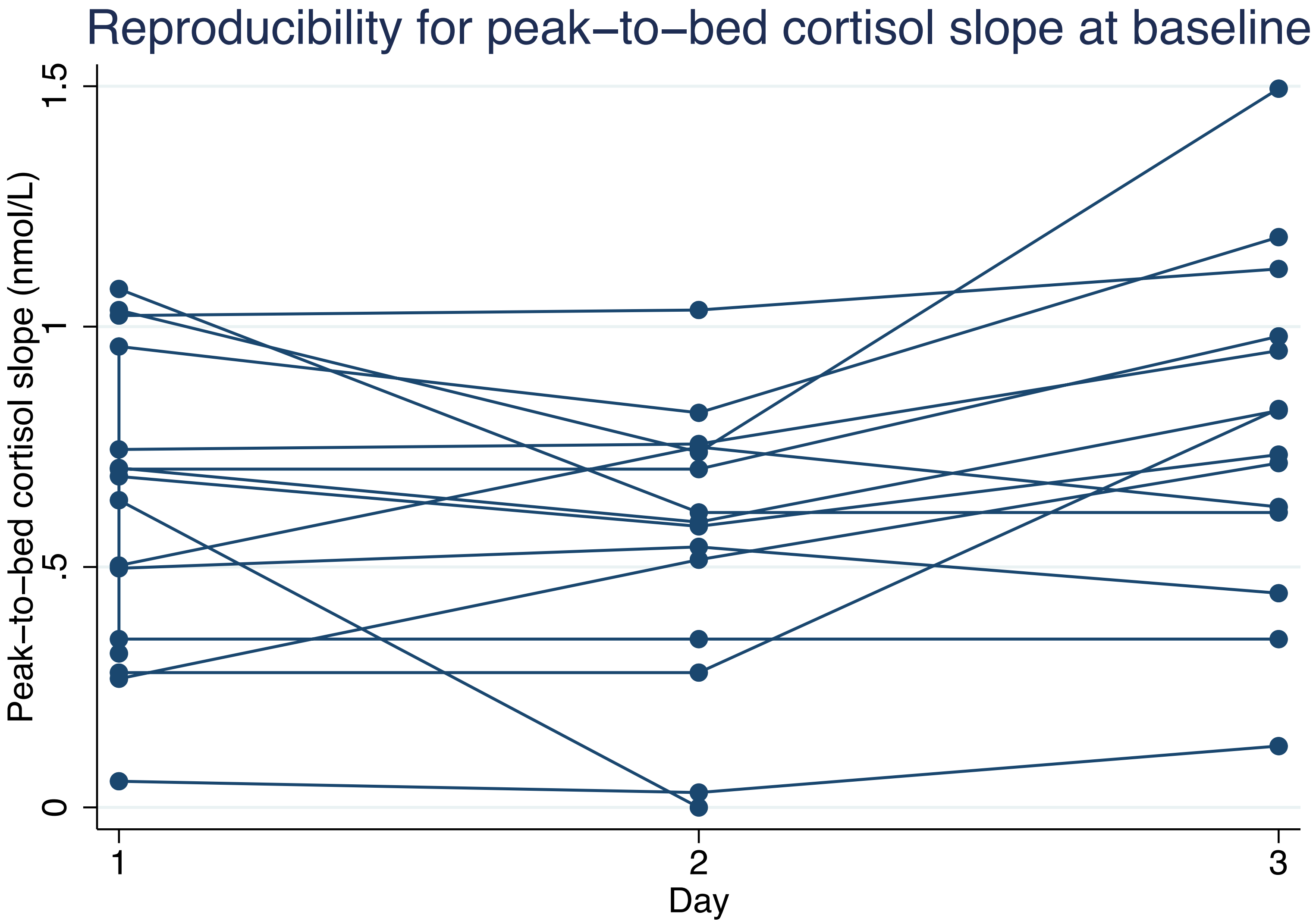

Reproducibility for peak–to–bed cortisol slope at baseline
Peak–to–bed cortisol slope (nmol/L)
0
.5
1
1.5
1
2
3
Day

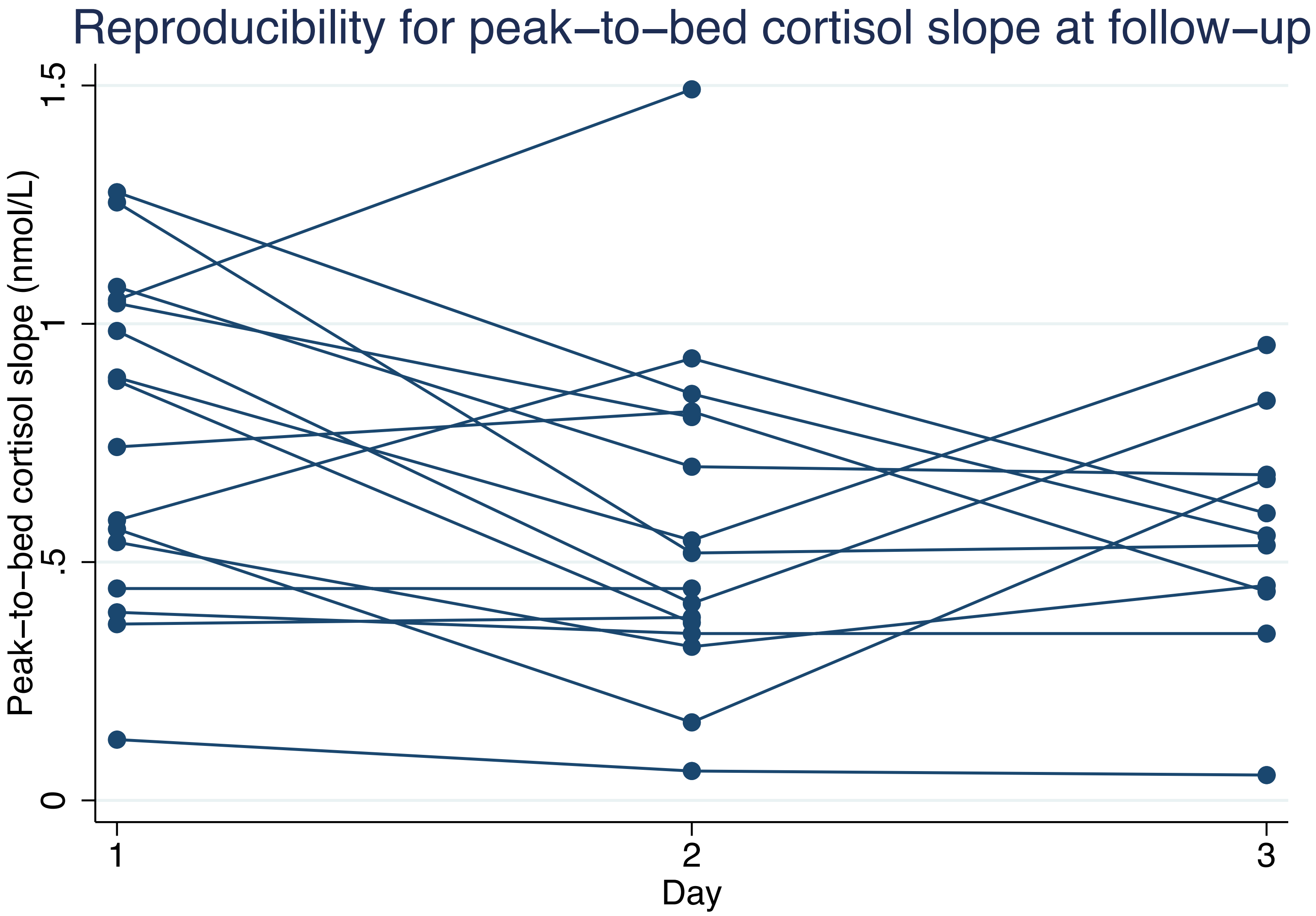

Reproducibility for peak–to–bed cortisol slope at follow–up
Peak–to–bed cortisol slope (nmol/L)
0
.5
1
1.5
1
2
3
Day
