# Supplementary material for: Feasibility of home-based sampling of salivary cortisol and cortisone in healthy adults
Source: BMC Res Notes. 2021 Nov 2;14:406. doi: 10.1186/s13104-021-05820-4 (PMC8561883; doi:10.1186/s13104-021-05820-4)
Supplement: Supplementary file 9 — Additional file 9. Intraclass correlation coefficients (ICC) for different cortisol and cortisone measurements and required measuring day based on follow-up values. The table present the intraclass correlation coefficient (ICC) for concentrations of cortisol and cortisone within the same participant and the required number of measuring days to obtain ICC values of 0.60, 0.70 and 0.80. [file 13104_2021_5820_MOESM9_ESM.docx]

## Additional file 9: Intraclass correlation coefficients (ICC) for different cortisol and cortisone measurements and required measuring day based on follow-up values

|  | ICC | Days to obtain | Days to obtain | Days to obtain |
| --- | --- | --- | --- | --- |
|  |  | ICC = 0.60 | ICC = 0.70 | ICC = 0.80 |
| Cortisol |  |  |  |  |
| S1 | 0.55 | 1 | 1 | 2 |
| CAR |  |  |  |  |
| CAR30 (30 minutes - S1) | 0.20 | 2 | 3 | 5 |
| CARpeak (Peak cortisol - S1) | 0.41 | 5 | 7 | 12 |
| CARauc | 0.60 | 1 | 1 | 2 |
| Diurnal cortisol slope |  |  |  |  |
| Wake-to-bed slope | 0.14 | 9 | 14 | 25 |
| Peak-to-bed slope | 0.55 | 2 | 3 | 5 |
| Cortisone |  |  |  |  |
| S1 | 0.32 | 3 | 5 | 8 |
| CAR |  |  |  |  |
| CAR30 (30 minutes - S1) | 0.53 | 1 | 2 | 4 |
| CARpeak (Peak cortisol - S1) | 0.19 | 6 | 10 | 17 |
| CARauc | 0.60 | 2 | 2 | 4 |
| Diurnal cortisol slope |  |  |  |  |
| Wake-to-bed slope | 0.28 | 4 | 6 | 10 |
| Peak-to-bed slope | 0.66 | 2 | 3 | 5 |
